# Mechanical properties of paraformaldehyde-treated individual cells investigated by atomic force microscopy and scanning ion conductance microscopy

**DOI:** 10.1186/s40580-017-0099-9

**Published:** 2017-03-20

**Authors:** Seong-Oh Kim, Joonhui Kim, Takaharu Okajima, Nam-Joon Cho

**Affiliations:** 1 School of Materials Science and Engineering, Nanyang Technological University, 50 Nanyang Avenue, Singapore, 639798 Singapore; 2 School of Chemical and Biomedical Engineering, Nanyang Technological University, 62 Nanyang Drive, Singapore, 637459 Singapore; 3 Graduate School of Information Science and Technology, Hokkaido University, Sapporo, 060-0814 Japan

**Keywords:** Scanning ion conductance microscopy, Atomic force microscopy, Cellular mechanics, Cell fixation

## Abstract

**Background:**

Cell fixation is an essential step to preserve cell samples for a wide range of biological assays involving histochemical and cytochemical analysis. Paraformaldehyde (PFA) has been widely used as a cross-linking fixation agent. It has been empirically recognized in a gold standard protocol that the PFA concentration for cell fixation, *C*_PFA_, is 4%. However, it is still not quantitatively clear how the conventional protocol of *C*_PFA_ is optimized.

**Methods:**

Here, we investigated the mechanical properties of cell fixation as a function of *C*_PFA_ by using atomic force microscopy and scanning ion conductance microscopy. The goal of this study is to investigate the effect of *C*_PFA_ (0–10 wt%) on the morphological and mechanical properties of live and fixed mouse fibroblast cells.

**Results:**

We found that both Young’s modulus, *E,* and the fluctuation amplitude of apical cell membrane, *a*_m_, were almost constant in a lower *C*_PFA_ (<10^−4^%). Interestingly, in an intermediate *C*_PFA_ between 10^−1^ and 4%, *E* dramatically increased whereas *a*_m_ abruptly decreased, indicating that entire cells begin to fix at *C*_PFA_ = ca. 10^−1^%. Moreover, these quantities were unchanged in a higher *C*_PFA_ (>4%), indicating that the cell fixation is stabilized at *C*_PFA_ = ca. 4%, which is consistent with the empirical concentration of cell fixation optimized in biological protocols.

**Conclusions:**

Taken together, these findings offer a deeper understanding of how varying PFA concentrations influence the mechanical properties of cells and suggest new avenues for establishing refined cell fixation protocols.

## Background

Understanding how cells behave at material interfaces holds wide importance for key biological applications such as cell–material surface interactions [1], mechanobiology [2], and advanced cell analysis [3]. Among such applications, one of the most practical and widely methods used across the biological sciences is cell fixation, which is an essential process for histological analyses in clinical diagnosis. Typically, when cells are degraded or dehydrated, essential cell components, such as protein, membrane, and intracellular structures will also be altered or degraded [4]. The surface structure of these cells may also collapse and diffuse away during antibody incubation and washing steps. Cell fixation aims to maintain cells or cellular components in life-like state, preventing unexpected changes by preserving essential chemical and physical characteristics of cells for further observation. Furthermore, cell fixation provides an effective approach for immunostaining by allowing the antibodies to access intracellular structures [5].

Among various fixation agents for cross-linking cell membrane and cytoplasmic protein, paraformaldehyde (PFA) is one of the most widely used chemical agents for cell and tissue samples [4–6]. PFA causes covalent cross-links between molecules, effectively gluing them together into an insoluble meshwork that alters the mechanical properties of the cell surface. Previous studies report that the cell surface hardens after fixative treatment [7–10]. Compared to an unfixed cell, the mechanical properties of a fixed cell are more uniform across the entire cell surface [11]. However, there is no systematic assessment of correlation between changes in mechanical properties of live and fixed cells. Indeed, little is known about how the mechanical properties of cells depend on the PFA concentration. Furthermore, it has been revealed that subtle adjustment in fixation conditions with, e.g., PFA condition, can have dramatic effects on the immobilization of molecules within cellular membranes [12]. Understanding the detailed process of cell fixation in various states from living cells to completely fixed cells provides an opportunity to optimize cell fixation protocols and to gain useful knowledge about the living cell fixation process.

To address this outstanding question, we investigated the mechanical properties of cell surface structures as a function of the concentration of PFA (*C*_PFA_) by using atomic force microscopy (AFM) and scanning ion conductance microscopy (SICM). These methods allow us to measure the elastic modulus and the surface fluctuation amplitude, respectively, of cells in both living and fixed states [13–16]. These measurement approaches can be applied to living cells to investigate cell mechanical changes in response to the PFA concentration, *C*_PFA_. We found that both cell stiffness and cell surface fluctuation underwent a transition around *C*_PFA_ = 10^−1^–4%, and these quantities were unchanged at a higher *C*_PFA_ (>4%), indicating that the cell fixation is stabilized at *C*_PFA_ = ca. 4%, which is consistent with the empirical concentration of cell fixation optimized in biological protocols.

## Results and discussion

### Topographical imaging of live and fixed L929 cells with SICM

**Fig. 1:**
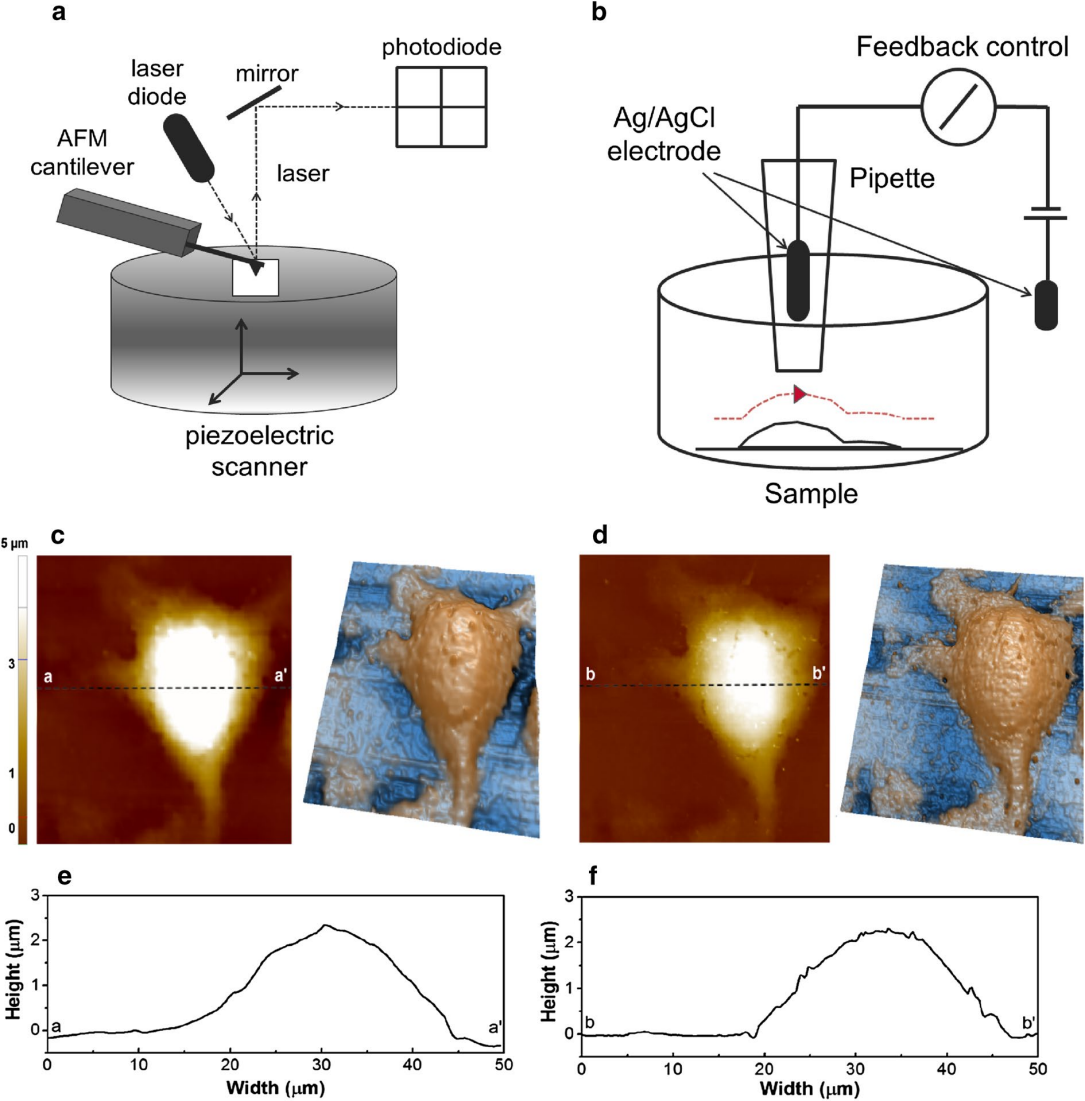
Schematic view of principle of SPM techiques and L929 cell surface images using SICM. **a** In AFM, the attractive or repulsive force between the tip and the sample causes deflection of the cantilever. As the cantilever deflects, the angle of the reflected laser beam changes angle and strikes a different part of the photodiode. The signals from the four quadrants of the detector are compared to calculate the deflection signal. Using this signal, the system (computer) generates a topography of the sample surface. **b** In SICM, a nano-pipette filled with electrolyte is brought in proximity to the sample of interest. A bias applied between an electrode in the pipette and another electrode in the bulk solution generates an ion current, which can be used in feedback control to prevent direct contact between the nano-pipette and the sample. **c** Height image and 3-dimensional image of live single L929 fibroblast cell surface using SICM hopping mode, **d** height image and 3-dimensional image of fixed fibroblast cell surface. The size of all images are 50 × 50 µm, after imaging of live cell (**c**), fixed with PFA, fixed cell imaging (**d**) was performed. **e**, **f** Indicates line profile of each image. Imaging time of each image is around 30 min

Figure 1c, d show the topography of a single L929 cell imaged by SICM before and after treating with 4% PFA, which is the conventional PFA concentration for cell fixation. Whereas no clear difference between the untreated and treated cells was observed for the cell height, a small difference in cell shape was apparent. The live cell shows a maximum height of 2.5 µm (Fig. 1e), and the corresponding height in the 4% PFA treated cell appears around 2.5 µm (Fig. 1f). However, the PFA-treated cells are slightly shrunken so that the cell adhesive area and the cell volume were lower than those of the untreated cell, which is in good agreement with anecdotal observations in the biology field [17]. The measured cell area and volumes are 908.12 µm^2^ and 774.11 µm^3^ in the live cell and 876.85 µm^2^ (3.5% lower) and 716.54 µm^3^ (7.4% lower) in 4% PFA treated cell. Furthermore, we found that the roughness of cell surface is larger in treated cells. This is probably due to crosslinking of proteins that react with PFA, resulting in aggregation and cell shrinkage.

### Surface fluctuations of cells with PFA treatment

**Fig. 2:**
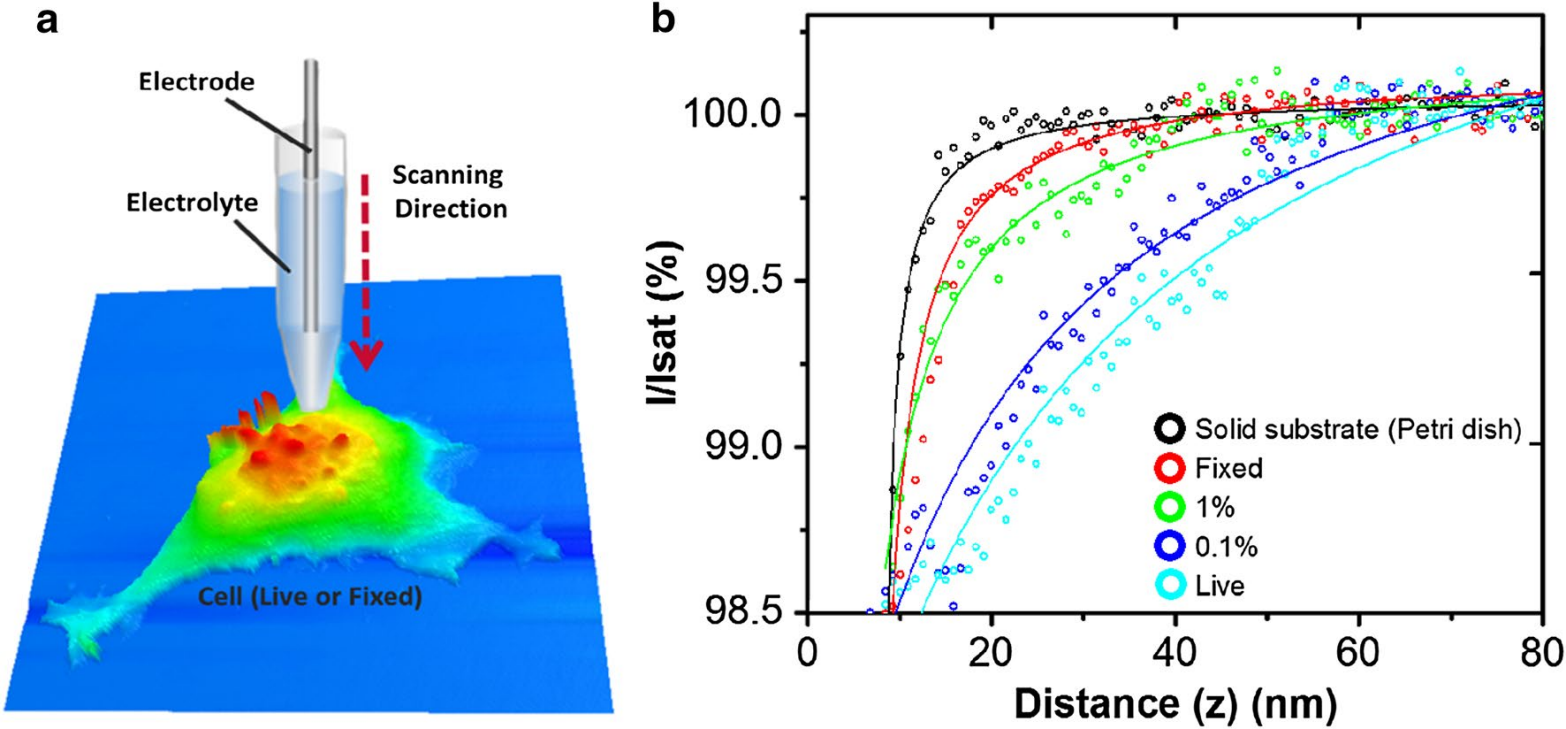
**a** Schematic view of the cell surface fluctuation setting by SICM. To investigate fluctuation levels, the nano-pipette was positioned on the apex of the single cell. **b** Typical ion current–distance curves of the solid substrate (*black*), a fixed cell (*red*), a 1% PFA treated cell (*green*), a 0.1% PFA treated cell (*blue*) and a live cell (*sky blue*). For the solid substrate (petri dish), there was no change. In the fixed cell, a much broader curve range was observed. For the live cell, the broadness of the curve range was greatest

**Fig. 3:**
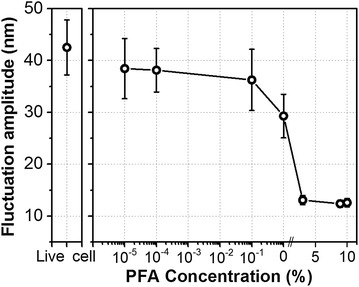
RMS displacements of cell surface fluctuations. The displacements are approximately 12 nm on a fixed cell, but much larger on a live cell surface (43 nm). The live cell shows more active movement than fixed cell (N = 60)

We measured the I–D curves of SICM as applied to cell surfaces at various *C*_PFA_. To estimate cell surface fluctuations, the measurement time of the I–D curve was checked against a known standard [16, 18, 19]. Figure 2b shows the representative I–D curves for L929 cells with different C_PFA_’s. In the solid substrate (petri dish), I–D curve exhibits the steepest slope, while in the untreated condition, I–D curve exhibits the broadest slope. In the PFA-treastd cells, the I–D curves exists in between. Note that the cell treated with 4% PFA was almost the same I–D curve as the solid substrate I–D curves. The I–D curves in Fig. 2b were fitted to Eq. (1), and the RMS displacement of cell surface fluctuations on treatment of different *C*_PFA_ are shown in Fig. 3. The estimated RMS displacement of surface fluctuations (a_m_) is approximately 12 nm on the 4% C_PFA_ treated cell. However, it increases gradually depending on the lower concentration of PFA. In the live cell, displacements was around 43 nm. The chemically fixation caused no difference on cell surface topography (Fig. 1e, f) however, the surface dynamics was drastically varied. It means that the live L929 cell had more active movement compare to the PFA treated cell. Smaller surface fluctuations in PFA treated cell can be explained by the PFA treatment effect that results in cross-linking of proteins between the membrane and cytoplasmic proteins.

### Young’s modulus of cells with PFA treatment

**Fig. 4:**
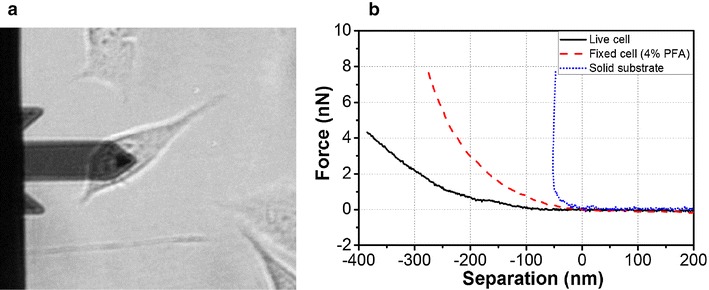
**a** Optical image of AFM tips positioning on the target cell. **b** Typical force-separation curve for a fixed cell (4% PFA-treated cell), live cell, and solid substrate. Compared to live cells, the slope of fixed cells approach curve is steeper, indicating a significantly larger Young’s modulus for fixed cells

**Fig. 5:**
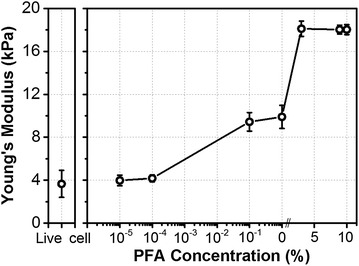
Young’s modulus for varying PFA concentrations in fibroblast cells. As expected, higher concentrations resulted in a higher Young’s modulus: approximately 3.5 kPa on live cells, and 18 kPa on fixed cells (N = 50)

To investigate the effect of PFA treatment on cell surface stiffness, the force-separation curve measurements were performed on live and PFA treated cells (Fig. 4a). Figure 4b shows typical force-separation curve for live cells, 4% PFA treated cells and solid substrate (petri dish). Clearly, the 4% PFA treated cell exhibits a much steeper force curve slope than a live cell. In addition, the required force for surface indentation is also larger for treated cells than for live cells. Figure 5 shows the averaged Young’s modulus, *E*, values as a function of C_PFA_. Compared to live cells (3.5 kPa), the stiffness of PFA treated cells (4% *C*_PFA_ treated cell is 18 kPa) gradually increases with higher *C*_PFA_.

The cell stiffness measured with AFM is strongly affected by the actin filamentous structures. PFA treatment affects the cross-linking of cell surface proteins, including F-actin filaments [5, 9, 17, 20, 21]. Taken together, we concluded that the stiffening of cells directly results in the cross-linking of proteins. Specifically, it is assumed that the PFA fixation caused an increase of the cell stiffness depending on the available number of randomly distributed crosslinking sites on the cell surface. As shown in Fig. 5, Young’s modulus does not increase linearly with the *C*_PFA_ which suggests saturation in the number of available sites for surface protein cross-linking. Below the 10^−1^% of *C*_PFA_ treatment, in lower concentration, there is no significant effect on cell stiffness, but it gradually increases because the crosslinking is occurred randomly. In the intermediate C_PFA_ (10^−1^%), a percolation of the crosslinking occurs, so that the stiffness undergoes a transition. When treated with higher concentrations of PFA (over 10^−1^%), it produces dramatic changes in cell stiffness. In this case, the stiffness remains unchanged, indicating the percolation is completed around C_PFA_ = 4%. Interestingly, the elastic modulus begins to increase before the membrane fluctuation amplitude decreases, suggesting that cells can tolerate some degree of protein cross-linking while maintaining normal function. Such findings are in excellent agreement with previous observations that molecules in cellular membranes can remain mobile under certain, relatively mild PFA fixation conditions [12].

**Fig. 6:**
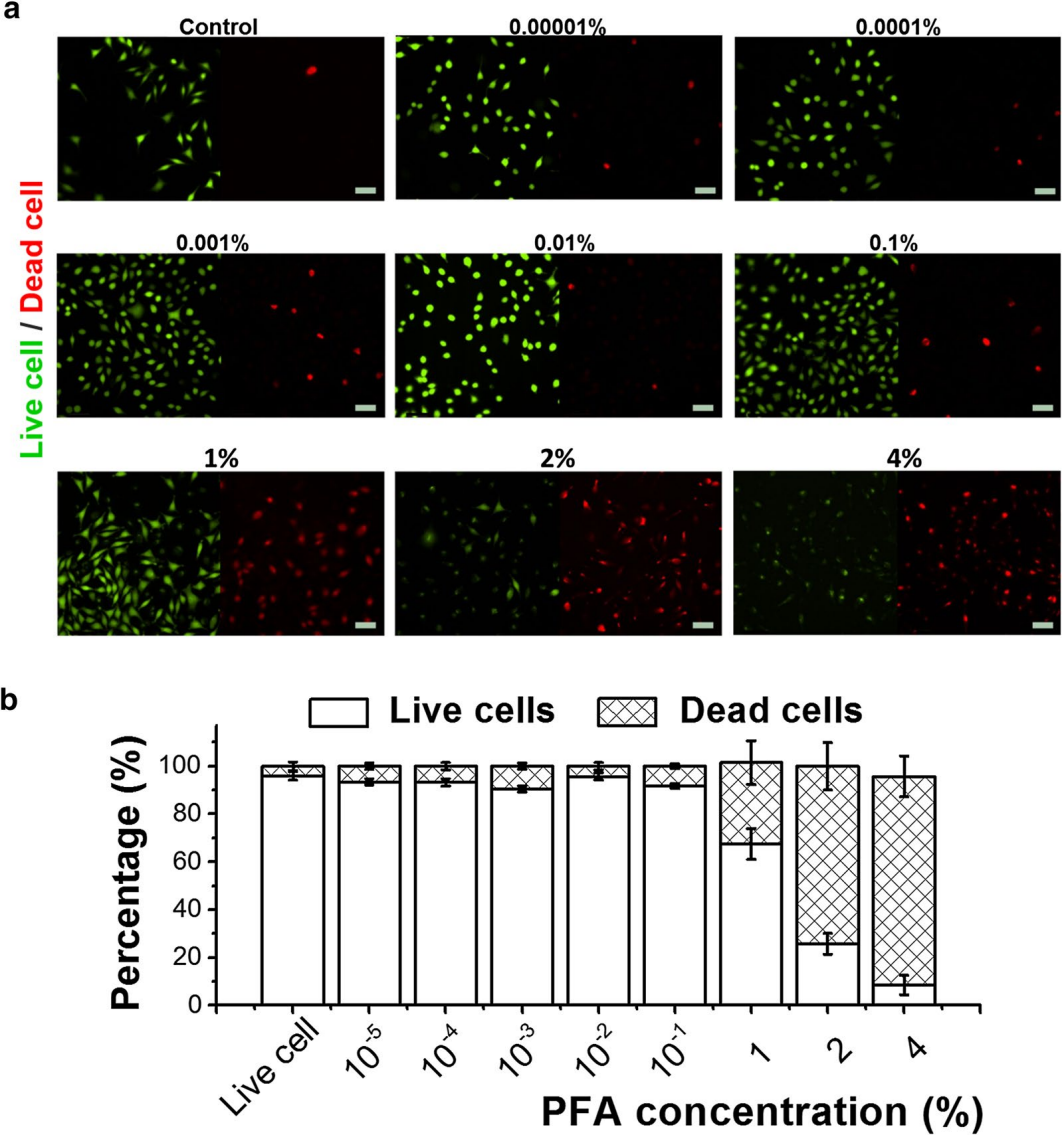
Evaluation of PFA-mediated cytotoxicity on L929 cells. **a** Fluorescence microscope images for assessment of live and dead cell ratio dependent on titration of *C*_PFA_. *Green fluorescence* represents live cells and *red fluorescence* represents dead cell, *scale bar* 50 μm. **b** Percentage graph of live/dead cell ratio dependent on the titration of *C*_PFA_. Data is presented as the mean ± standard deviation, with *t* test results indicating p < 0.05

To corroborate these findings, cell viability experiments were conducted at different PFA concentrations. Figure 6a shows live and dead cells as distinguished via the staining kit. Green color represents live cells, and red color represents dead cells. As shown in this figure, from control to 0.1% PFA treatment, most of the cell culture is live. PFA treatment over 1% shows a large increase in red cells, indicating widespread cell death. Also, Fig. 6b shows the quantitative analysis of live and dead ratio from staining data after PFA treatment. Between 0.1 and 1% *C*_PFA_ appears to be the critical concentration for live cell to dead cell ratio inversion. This agreement between cell surface fluctuation, Young’s modulus and cell viability assay data supports the statement that range encompassing 0.1–1% *C*_PFA_ can trigger sufficient surface protein cross-linking, and in particular that a critical density of cross-linking events occurs in this PFA concentration range whereby cellular function is irreversibly impaired and likely related to changes in membrane mobility as well. In other words, this PFA concentration range is critical for to influencing the available number of crosslinking sites on the cell surface, and with that, cell viability.

## Conclusions

Herein, we have demonstrated a fundamental mechanical comparison between live cells and cells that were fixed with various concentrations of PFA. AFM and SICM measurements showed that the apparent surface fluctuation amplitude and elastic modulus of cells underwent transition when exposed to PFA concentrations between 0.1 and 4%. After complete PFA fixation, cell surface fluctuation decreased to 71% of live cell, while the Young’s modulus increased by fivefold compared to that of live cells. These results provide a deeper understanding of how cells react to chemical treatment with PFA that takes into account not only the traditional chemical understanding of PFA’s effect upon the cell, but now also the cell’s surface-based mechanical properties that were targeted in this study. It is now apparent that PFA fixation enables the opening of distributed proteins across the cell surface, a critical process that facilitates widespread crosslinking. Cell membranes that are typically flexible and variable. But in a certain situation, such as chemical treatment, biological functions are changed, and morphological changes also occur. This is the reason why studying cell surface fluctuations are crucial for the understanding of cell function about cell dynamics. Given the general nature of these physicochemical mechanisms, we expect that similar effects of PFA treatment on the elastic modulus and membrane fluctuations would also be expected although the specific magnitudes and responses conferred upon PFA treatment might vary on an absolute scale. We have confidence in that the SPM techniques could well serve as a promising tool for quantitative studies of both fixed cells and live cells in order to further explore this exciting topic at the convergence of biology and nanotechnology.

## Methods

### Cell sample

We used mouse fibroblast L929 cells (ATCC, USA) cultured in Dulbecco’s modified eagle medium (DMEM; Invitrogen Life Technique, US) supplemented with 10% fetal bovine serum (Thermo Fisher Scientific, US) and 1% penicillin/streptomycin (Invitrogen Life Technique, USA) at 37 °C in a humidified atmosphere containing 5% CO_2_. The cell samples with cell densities of 1 × 10^4^/mL on a 35 mm diameter cell culture petri dish (NUNC, Denmark), were washed with phosphate buffered saline (PBS, Sigma-Aldrich, US) three times and then treated with different PFA solutions (*C*_PFA_ = 10^−5^, 10^−4^, 10^−1^, 1, 4, 8 and 10%) for 5 min. Before AFM and SICM experiments, the treated cell samples were again washed three times with PBS.

### SPM apparatus

A commercial SPM system (NX-Bio, Park Systems, South Korea) equipped with an inverted optical microscopy (Nikon Corp., Japan) which is designed specifically for biological applications was employed in this study. The SPM system not only achieved a soft material sample such as cell surface information using SICM, but it also obtained mechanical properties of a sample using AFM. All experiments using live cells were performed in a customized live cell chamber (Live Cell Instrument, South Korea). The live cell chamber was adjusted to 37 °C with 5% CO_2_ and 95% humidity, readily providing the specific environmental conditions needed to sustain cell cultures. Within this environment, live cell SICM imaging or AFM experiment was conducted for extended timeframes via optical monitoring methods, including optical phase contrast and digital image correlation microscopy.

#### SICM measurement for cell imaging and fluctuation analysis

The operation of SICM relies on an ion current that flows between an electrode inside a nano-pipette and an electrode located in an external bath solution. This ion current provides a feedback signal used to maintain the tip-sample distance and allow for the nano-pipette to scan topographical information (Fig. 1b). In spite of low lateral resolution (10–20 nm) [22], SICM offers useful topographical measurement without applying any mechanical force onto the sample surface.

SICM imaging and ion current-distance (I–D) curve experiments were performed using customized SPM system with 100 nm inner diameter nano-pipette fabricated from borosilicate capillaries (inner diameter 0.6 mm, outer diameter 1.0 mm, World Precision Instruments, USA) using a CO_2_-laser pipette puller (Sutter Instruments, USA). The cell topographic images were obtained with the so-called hopping mode [23], in which the nano-pipette approached sample surfaces with the pre-set threshold of 1.2%.

The apparent fluctuation amplitude of cell apical surfaces, *a*_m_, was estimated from the I–D curve of SICM measurement [16, 19]. The measured ion current with cell apical surface fluctuation was assumed to be a convolution of the non-fluctuation-based ion-current relation, *I*_0_, and the existing probability of cell surface position at z, *P*(*z*).

1 $$\left\langle I( {z - z_{0} ,\delta z^{2}_{s} }) \right\rangle = \mathop \smallint \limits_{ - \infty }^{\infty } I_{0} \left( {z - z_{0} } \right) P\left( {z_{s} - z_{0} , \delta z^{2}_{s} } \right) {\text{d}}z_{s}.$$

The *z* and *z*_*s*_ are the position of the pipette and the sample, respectively. The $z_{0}$ and $\delta z^{ 2}_{s}$ are the time-average position of cell surface and the deviation of the sample fluctuation. The non-fluctuation ion-current relation is approximately expressed as the following form [19]:

2 $$I_{0} \left( {z - z_{0} } \right) = I_{sat} \left( {1 + \frac{\zeta }{{z - z_{0} }}} \right)^{ - 1} ,$$

where *I*_*sat*_ is the reference current when the pipette is far enough from the sample surface, and *ζ* is a constant from the pipette geometry. It is here assumed that the cell fluctuation obeys the Gaussian distribution,

3 $$P\left( {z_{s} - z_{0} , \delta z^{2}_{s} } \right) = \frac{1}{{\delta z_{s} \sqrt {2\pi } }}\exp \left( { - \frac{1}{2}\left( {\frac{{z_{s} - z_{0} }}{{\delta z_{s} }}} \right)^{2} } \right).$$

The I–D curves measured at around the cell center were fitted to the Eq. (1) to estimate the apparent fluctuation deviation $\delta z^{ 2}_{s}$ $I_{sat}$ and $\zeta$ were determined experimentally to be $I_{sat}$ = 1 nA and $\zeta$ = 4.9 × 10^−2^ nm, respectively, which were estimated from the SICM measurement on a glass substrate [16]. According to Eq. (3), the fluctuation amplitude of apical cell membrane, *a*_m_ is defined as the Gaussian distribution, *P*, with the root mean square (RMS) displacement of cell surface fluctuation, $\langle \delta z^{2}_{s} \rangle^{{\frac{1}{2}}}$.

#### AFM measurements for Young’s modulus of cells

The force curve measurements of AFM were performed to estimate Young’s modulus of cells. We used a commercial AFM cantilever (Biolever mini, Olympus, Japan) with less than 0.09 N/m of a nominal spring constant. Because a cantilever with a small spring constant makes a relatively large deflection for a small force, the cantilever used in this study provides reliable data of the cell surface structure. The spring constant of the AFM cantilever was calibrated using the thermal vibration method [24]. AFM cantilever was cleaned using ethanol and exposed to UV light for 30 min to remove contamination on the AFM cantilever and tip. We measured more than 50 force curves with 512 data points. The force curves were analyzed with a Hertz model using a commercial SPM data analysis program (Park Systems, South Korea). We assumed the AFM tip shape is four-sided pyramid with a half cone angle *α*, so that the force on cantilever *F* is expressed as.

4 $$F = \frac{E}{{1 - v^{2} }}\frac{\tan \alpha }{\sqrt 2 }\delta^{2} ,$$

where *E* is the Young’s moduls, *ν* is the Poisson’s ratio and *δ* is the indentation (depth). *ν* and alpha were set to be 0.5° and 35°, respectively. The scan rate of the AFM cantilever and the maximum loading force were set to be 1–2 µm/s and 3–8 nN, respectively.

### Cell viability assay

To evaluate the viability of cells with PFA treatment, we used a LIVE/DEAD^®^ Viability/Cytotoxicity Kit (L3224; Invitrogen life technique, USA). Briefly, the PFA-treated cells were immediately incubated using the live and dead stain fluorescence dye for 10 min. Then the final 2 µM calcein AM and 4uM EtD-1 mixture solution were added to the PFA-treated cell sample. A commercial fluorescence microscope (Nikon Corp., Japan) was used to obtain fluorescence images of cells where green and red colors represented live and dead cells, respectively.
